# Retracted: Obesity as a Consequence of Gut Bacteria and Diet Interactions

**DOI:** 10.1155/2015/786924

**Published:** 2015-03-12

**Authors:** International Scholarly Research Notices

The paper titled “Obesity as a Consequence of Gut Bacteria and Diet Interactions” [1], published in International Scholarly Research Notices, has been retracted, upon the authors' request, as it is essentially identical in content with a previously published paper by the same authors titled “Bacteria and Obesity: The Proportion Makes the Difference,” published in Surgery: Current Research (2013) 3:152.
